# Imaging the development of the human craniofacial arterial system – an experimental study

**DOI:** 10.1007/s00247-024-06044-x

**Published:** 2024-09-10

**Authors:** K. Jacobs, G. E. J. Langenbach, D. Docter, P. A. M. Cordewener, B. J. van de Beek, J. A. M. Korfage, S. C. Visser, J. J. Peters, J. Hagoort, F. Lobbezoo, B. S. de Bakker

**Affiliations:** 1 Department of Oral Pain and Disfunction, Section Orofacial Anatomy, Academic Centre for Dentistry Amsterdam (ACTA), University of Amsterdam and VU University Amsterdam, Gustav Mahlerlaan 3004, 1081LA Amsterdam, The Netherlands; 2 Department of Medical Biology, Section Clinical Anatomy & Embryology, Amsterdam UMC, location AMC, Meibergdreef 15, 1105AZ Amsterdam, The Netherlands; 3 Amsterdam Reproduction and Development Research Institute, Meibergdreef 9, 1105AZ Amsterdam, The Netherlands; 4 Department of Plastic, Reconstructive and Hand Surgery, Amsterdam UMC, location AMC, Meibergdreef 9, Amsterdam, The Netherlands; 5 Department of Obstetrics and Gynecology, Amsterdam UMC, location AMC, Meibergdreef 9, Amsterdam, The Netherlands; 6 Department of Pediatric Surgery, Erasmus MC – Sophia Children’s Hospital, University Medical Center Rotterdam, Dr. Molewaterplein 40, Rotterdam, The Netherlands; 7 Department of Pediatric Surgery, Emma Children’s Hospital, Amsterdam UMC, location AMC, Meibergdreef 9, Amsterdam, The Netherlands; 8 Amsterdam Gastroenterology Endocrinology Metabolism, Meibergdreef 9, Amsterdam, The Netherlands

**Keywords:** Craniofacial abnormalities, Embryology, Micro-computed tomography, Microvasculature, Vascular development

## Abstract

**Background:**

The process of vascular development is essential for shaping complex craniofacial structures. Investigating the interplay between vascular development and orofacial morphogenesis holds critical importance in clinical practice and contributes to advancing our comprehension of (vascular) developmental biology. New insights into specific vascular developmental pathways will have far-reaching implications across various medical disciplines, enhancing clinical understanding, refining surgical techniques, and elucidating the origins of congenital abnormalities. Embryonic development of the craniofacial vasculature remains, however, under-exposed in the current literature. We imaged and created 3-dimensional (D) reconstructed images of the craniofacial arterial system from two early-stage human embryonic samples.

**Objective:**

The aim of this study was to investigate the vascular development of the craniofacial region in early-stage human embryos, with a focus on understanding the interplay between vascular development and orofacial morphogenesis.

**Materials and methods:**

Reconstructions (3-D) were generated from high-resolution diffusible iodine-based contrast-enhanced computed tomography (diceCT) images, enabling visualization of the orofacial arterial system in human embryonic samples of Carnegie stages (CS) 14 and 18 from the Dutch Fetal Biobank, corresponding to weeks 7 and 8.5 of gestation.

**Results:**

From two human embryonic samples (ages CS 14 and 18), the vascular development of the orofacial region at two different stages of development was successfully stained with B-Lugol and imaged using a micro-computed tomography (micro-CT) scanner with resolutions of 2.5-μm and 9-μm voxel sizes, respectively. Additionally, educational 3-D reconstructions of the orofacial vascular system were generated using AMIRA 2021.2 software.

**Conclusion:**

Micro-CT imaging is an effective strategy for high-resolution visualization of vascular development of the orofacial region in human embryonic samples. The generated interactive 3-D educational models facilitate better understanding of the development of orofacial structures.

**Graphical abstract:**

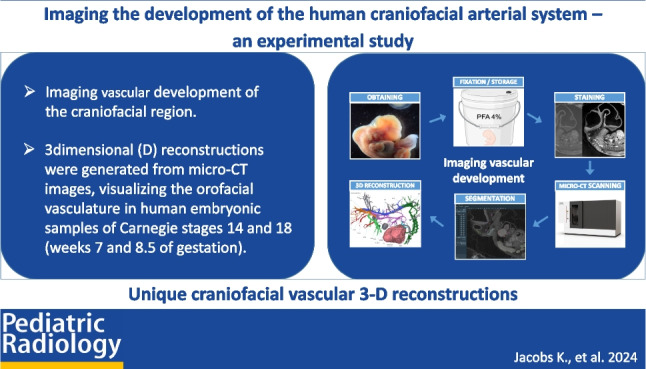

**Supplementary information:**

The online version contains supplementary material available at 10.1007/s00247-024-06044-x.

## Introduction

The complex process of vascular development plays a pivotal role in shaping the intricate craniofacial structures [1]. Proper vascular development is paramount for normal growth and maturation of tissues [2, 3]. In comparison with the extensive research conducted in the realm of neurovascular development, our understanding of orofacial vascular development remains limited. Orofacial malformations, such as craniofacial synostoses, cleft lip and cleft palate, micrognathia, and macroglossia, pose significant challenges in clinical practice [4]. Despite their prevalence and clinical significance, the etiology of many of these malformations remains elusive. This knowledge gap underscores the need to delve deeper into the role of vascular development in the genesis of craniofacial malformations. By unraveling the complexities of craniofacial vascular development, we aim to shed light on the underlying mechanisms contributing to a spectrum of craniofacial malformations [1].

### Historical perspective of imaging human development

Traditionally, the majority of developmental research focused on visualizing and describing vascular development relied on histological sections of embryologic specimens for analysis and reconstruction. In this technique, individual surfaces, obtained from these sections, generated sequentially using a microtome, were reconstructed to form a 3-dimensional (D) visualization [5]. This technique might be considered the gold standard for visualizing embryological development. Wilhelm His, a prominent anatomist and embryologist (1831–1904), adopted this method in his embryological research [6]. Initially focusing on chick embryos, he later applied microtome techniques to human embryos, producing histological stained tissue sections for reconstructions of, what Wilhelm His named, “plastic views” of entire embryos or specific organs [6]. His work introduced human specimens to the research of embryological development during the late nineteenth century. Employing samples derived from hysterectomies, ectopic pregnancies, and spontaneous abortions, obtained through a network of medical professionals, he aimed to standardize developmental depictions of human embryos, which were then termed “normal plates” [7, 8]. More recently, Rana et al. [9] created a 3-D reference model from the aortic arch development using a digital version of the similar techniques. Recently, Belle et al. [10] published their remarkable study employing light sheath microscopy as a modality for imaging and 3-D visualization of detailed human embryological development at various stages. For imaging vascular development, light sheath microscopy has been proven as an outstanding technique, providing excellent details and 3-D visualization of the developing vascular tree [11, 12]. In addition, Ramirez et al. [12] showed promising results visualizing and analyzing pharyngeal arch arteries with an immunohistochemistry technique and 3-D reconstructions. However, a great disadvantage of using these techniques on precious embryonic samples is their destructive character.

Fortunately, new technological modalities have been developed, such as computed tomography (CT), various microscopy techniques, and ultrasound, capable of visualizing structures and making 3-D reconstructions without destructing the original sample. In recent years, it is demonstrated that advancements in imaging techniques have revolutionized our ability to investigate intricate processes of embryonic development [10, 13]. The use of micro-CT imaging of human embryonic samples has emerged as a powerful tool for visualizing and analyzing the dynamic changes in vascular networks during orofacial development. This imaging modality allows for high-resolution, 3-D reconstruction of vascular structures, providing unprecedented insights into the spatial and temporal dynamics of vascular morphogenesis of the orofacial region [13, 14].

### Vascular development

The vascular development of the external carotid system starts with the initiation of the primary system for the extra cranial arteries at Carnegie stage (CS) 8 and finalizes at CS 23 [15, 16]. The majority of the vascular system, especially the major blood vessels of the body and the intracranial region, originates through vasculogenesis, a process involving the de novo formation of blood vessels [17]. In contrast, the development of extracranial vessels does primarily occur through angiogenesis, a process driven by hypoxia, and characterized by the emergence of new blood vessels through sprouting and branching from pre-existing ones [18, 19].

The primary aim of this study is to investigate the craniofacial vascular development in human embryos. To achieve this, we provide a comprehensive understanding on embryological development of the craniofacial arterial development. Additionally, we aim to produce educational 3-D reconstructions that will facilitate the comparison of our findings on early-staged craniofacial vascular development with the reviewed literature. By integrating these detailed reconstructions with the current body of knowledge, we hope to enhance the comprehension of craniofacial vascular anatomy, particularly focusing on the external carotid system. Such an enriched understanding is crucial for accelerating the identification of etiological factors underlying craniofacial birth defects.

## Methods

### Embryonic samples

#### Sample acquisition

In this study, two human embryonic samples were used: one at CS 14 (TOP 174, 4-mm crown-rump length (CRL)) and the other at CS 18 (TOP 318, 15-mm CRL), corresponding to gestational ages of 7 weeks and 8.5 weeks, respectively. These specific samples were included due to their availability from the Dutch Fetal Biobank [14]. The specimens were donated with parental written informed consent after removal of an ectopic pregnancy. Ethical approval of the Dutch Fetal Biobank was granted by the Medical Ethical Committee (METC) of the Amsterdam University Medical Center, location AMC, Amsterdam, The Netherlands (METC 2016_285).

#### Embryo sample preparation and scanning protocol (Fig. 1)

**Fig. 1:**
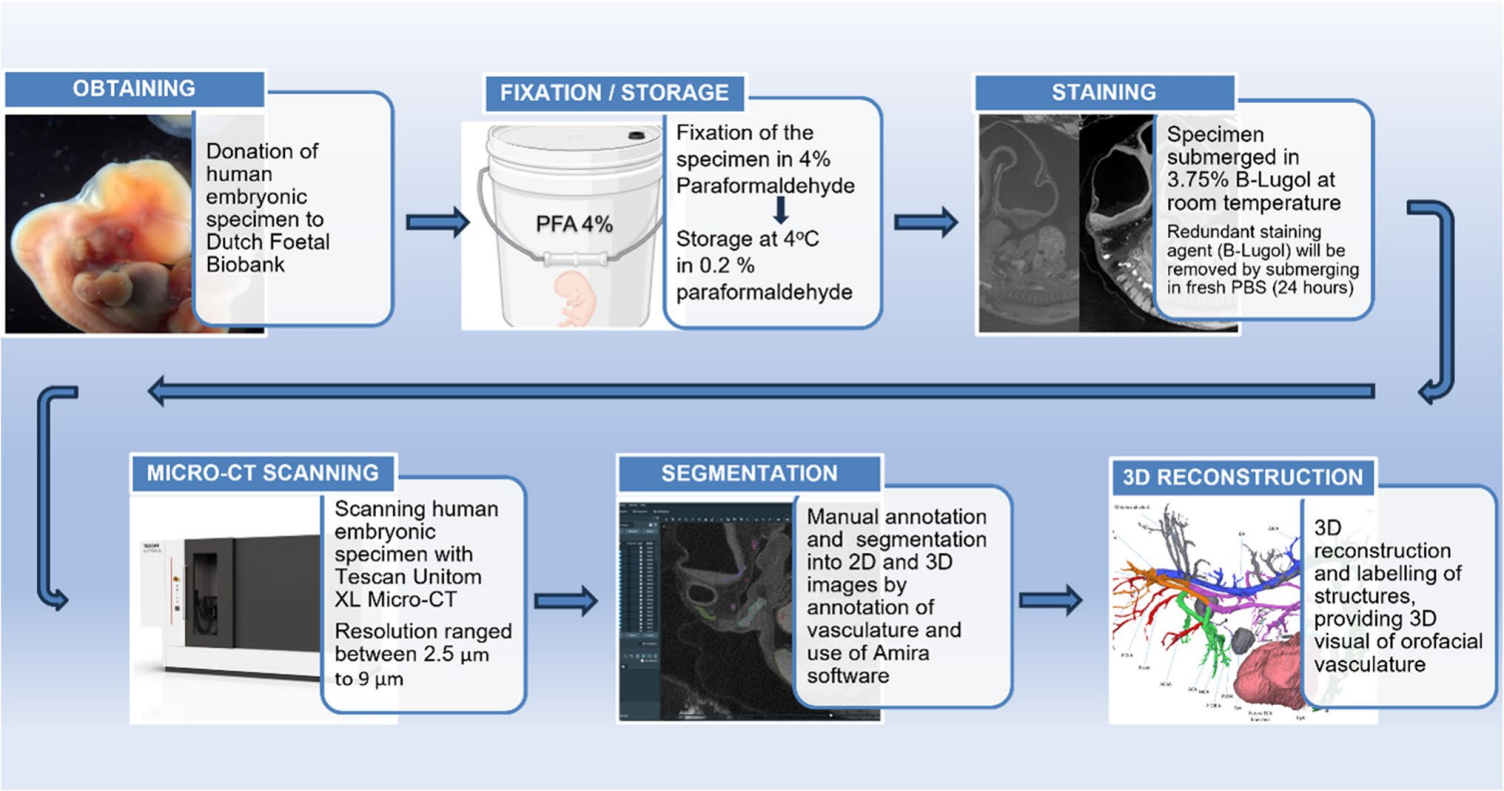
Graphical visualization of the workflow of data acquisition, demonstrating each step of the process: obtaining, fixation/storage, staining, micro-computed tomography scanning, segmentation, 3-dimensional reconstruction

After surgical resection of the fallopian tube, samples were fixed in 4% paraformaldehyde solution for 48 h, and then stored at 4 °C in a 0.2% paraformaldehyde solution. A diffusible iodine-based contrast-enhanced computed tomography (diceCT) staining technique for imaging of the human embryonic soft tissues was used [13, 14], prior to exposure of the embryo to the micro-CT scan. Staining was performed by submerging the sample for 5 days in a 3.75% buffered Lugol’s solution [20]. Apart from its general contrast-enhancement of tissues, this solution typically enhances the visibility of blood and vessels on micro-CT scans due to its high affinity with blood. Micro-CT scans were performed using a UniTOM XL micro-CT unit (TESCAN XRE Demo Lab, Ghent, Belgium) providing images with a voxel size for samples 1 (CS 14) and 2 (CS 18) of 2.5 μm (scan settings 80 kV, 15 W and scan time 7 h 42 min) and 9.0 μm (scan settings 100 kV, 15 W and scan time 2 h 16 min), respectively.

#### Data visualization

The software package Amira 3-D (version 2021.2, Thermo Fisher Scientific, MA, USA) was used for the creation of 3-D reconstructions of the intra- and extracranial vascular system [21]. Micro-CT data sets from both human embryonic samples (CS 14 and 18) were used for annotation and segmentation. The reconstructions were mostly manually segmented with a Bamboo tablet and stylus (http://www.wacom.com). After generation of the 3-D models, surfaces were smoothed, without loss of essential details. Labels were assigned to the different anatomical structures after the 3-D reconstructions were completed (Fig. 1). Segmentation and labeling were accomplished by two trained dentistry students, under the supervision of the first author.

Supplementary 3-D-PDFs containing the 3-D reconstructions of both stages were added to the online version of this article and can be viewed with a recent version of Adobe Reader (free-ware available at http://www.adobe.com).

### Terminology

#### Weeks/days

Gestational age, calculated from the first day of the last menstrual period, is commonly used to denote the clinical age of an embryo or fetus, while post-conception age refers to the number of days elapsed since fertilization. Throughout our study, we will refer to “days” or “weeks” as gestational age.

#### CS

The Carnegie stages, proposed by Streeter in 1942 and later revised by O’Rahilly and Müller in 1987 and 2010 [22], are based on the observed external features of developing human embryos in the first 60 days of their development post-fertilization.

### Background

The development of the craniofacial arterial system begins with the genesis of five pairs of primitive pharyngeal arch arteries, which undergo significant modifications throughout development [9, 15, 23–27]. The fundamental organization of the pharyngeal regions arises with the emergence of pharyngeal pouches, marking a crucial event in the development of the pharyngeal arches [23, 24]. Each pharyngeal arch contains various embryonic cell types, including ectoderm (external), endoderm (internal), and a mesenchymal filling of the neural crest with a central core of mesoderm [23]. These distinct embryonic cell populations contribute to different components of the arch: the ectoderm forms the epidermis and sensory neurons of the epibranchial ganglia; the endoderm gives rise to the epithelial lining of the pharynx, taste buds, thyroid, parathyroid, and thymus; the neural crest in this layer forms the skeletal and connective tissues of the arches; and the mesoderm contributes to the musculature and endothelial cells [23, 28].

During CS 11 to 20, the intracranial vascular system, including the circle of Willis, is formed. This process has been thoroughly studied and documented in the literature [16, 26, 27, 29], particularly due to its clinical significance and the association between congenital arterial malformations in intracranial vessels and the risk of cerebrovascular pathology later in life [30, 31].

Older pioneers in vascular development, as noted by Bertulli and Robert [15], have focused more on the formation of the extracranial arterial system. Notably, several studies by Streeter, Congdon, and Padget [16, 32–34] utilized the Carnegie embryo collection to outline the foundational principles of craniofacial vascular system formation. Additionally, significant contributions have been made by Altmann and Lasjaunias [35–37], who provided research on arterial variants, comparative anatomy, neurovascular anatomy, and embryology.

The development of the extracranial arterial system commences at CS 8, with the production of mesodermal substrate during the process of gastrulation. Subsequently, various modification and formation processes occur throughout embryonic and fetal development, ultimately culminating in the formation of the extracranial vascular system. Table 1 provides a schematic overview of these developmental stages.

**Table 1:**
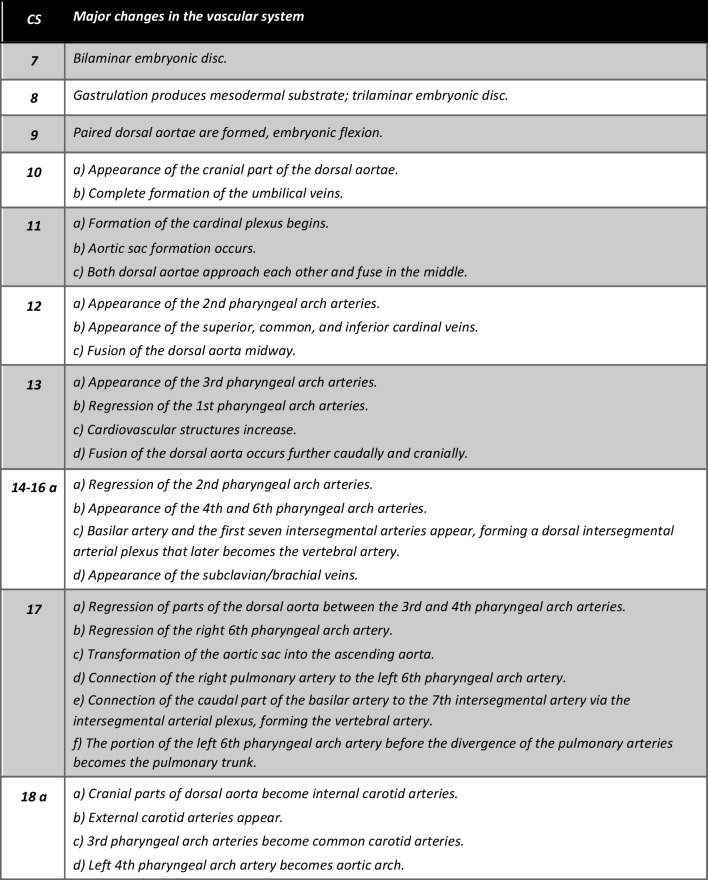
Overview showing developmental progression of the extracranial arterial system across Carnegie stages 7 until 23. From each stage, specific developmental processes are described, providing a comprehensive overview of this complex process

Adapted from De Bakker et al. 2016 [27], *a* In this study, we included two human embryonic samples corresponding to Carnegie stages 14 and 18

**Fig. 2:**
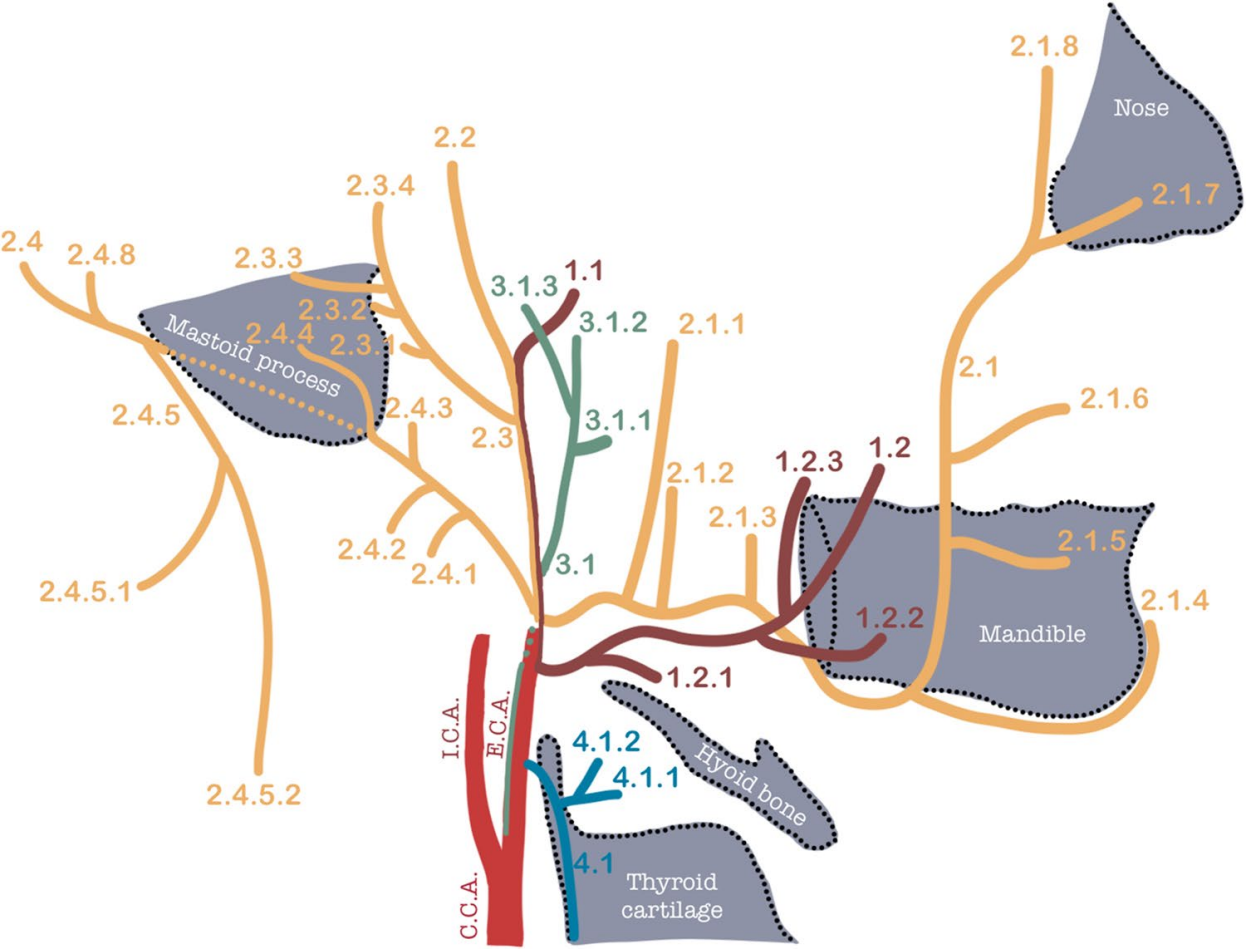
Graphical comprehensive overview of all arterial branches, their derivatives, and the corresponding pharyngeal arches of which they originate from, detailing the oxygenated blood supply to the orofacial and extracranial regions: 1st arch derivatives (*dark red*), 2nd arch artery derivatives (*orange*), 3rd arch artery derivatives (*green*), 4th arch derivatives (*blue*). *CCA* common carotid artery, *ECA* external carotid artery, *ICA* internal carotid artery (*bright red*). Details of each of these arteries are provided in Table 2 (Figure adapted from Cartens et al. and Thieme/Prometheus 2010 [38, 39])

Each of the pharyngeal arteries will differentiate, modulate, and finally participate in the development of specific parts of the extracranial vascular system and vascularize the orofacial and extracranial regions. The bilateral present external carotid artery (ECA) will be the basis of the extracranial vessels. Each of these vessels will find their origin in one of four different pharyngeal arches (1st–4th), with most of them deriving from the 2nd pharyngeal arch (see Fig. 2 with corresponding Table 2 for an overview of ECA branches and the pharyngeal artery they derived from).

**Table 2:**
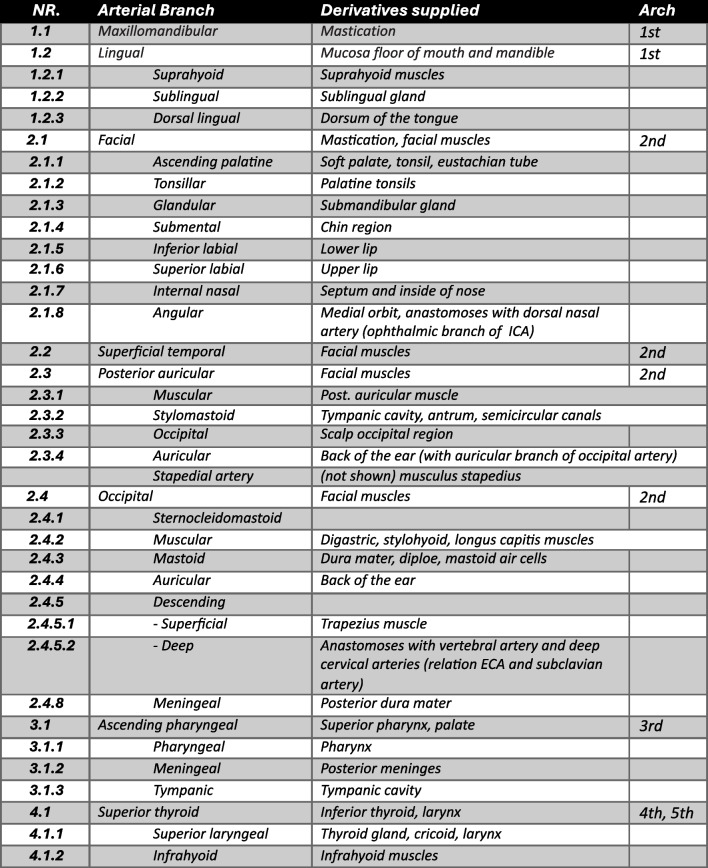
A comprehensive overview of all arterial branches, their derivatives, and the corresponding pharyngeal arches they originate from, detailing the oxygenated blood supply to the orofacial and extracranial regions, which corresponds to Fig. 2

Based on Thieme/Prometheus 2010: Atlas of Anatomy Head and Neck and Standrings *Gray’s anatomy* 2016 [39, 40]. *ECA* external carotid artery, *ICA* internal carotid artery

## Results

### Reconstruction embryonic sample 1, CS 14

**Fig. 3:**
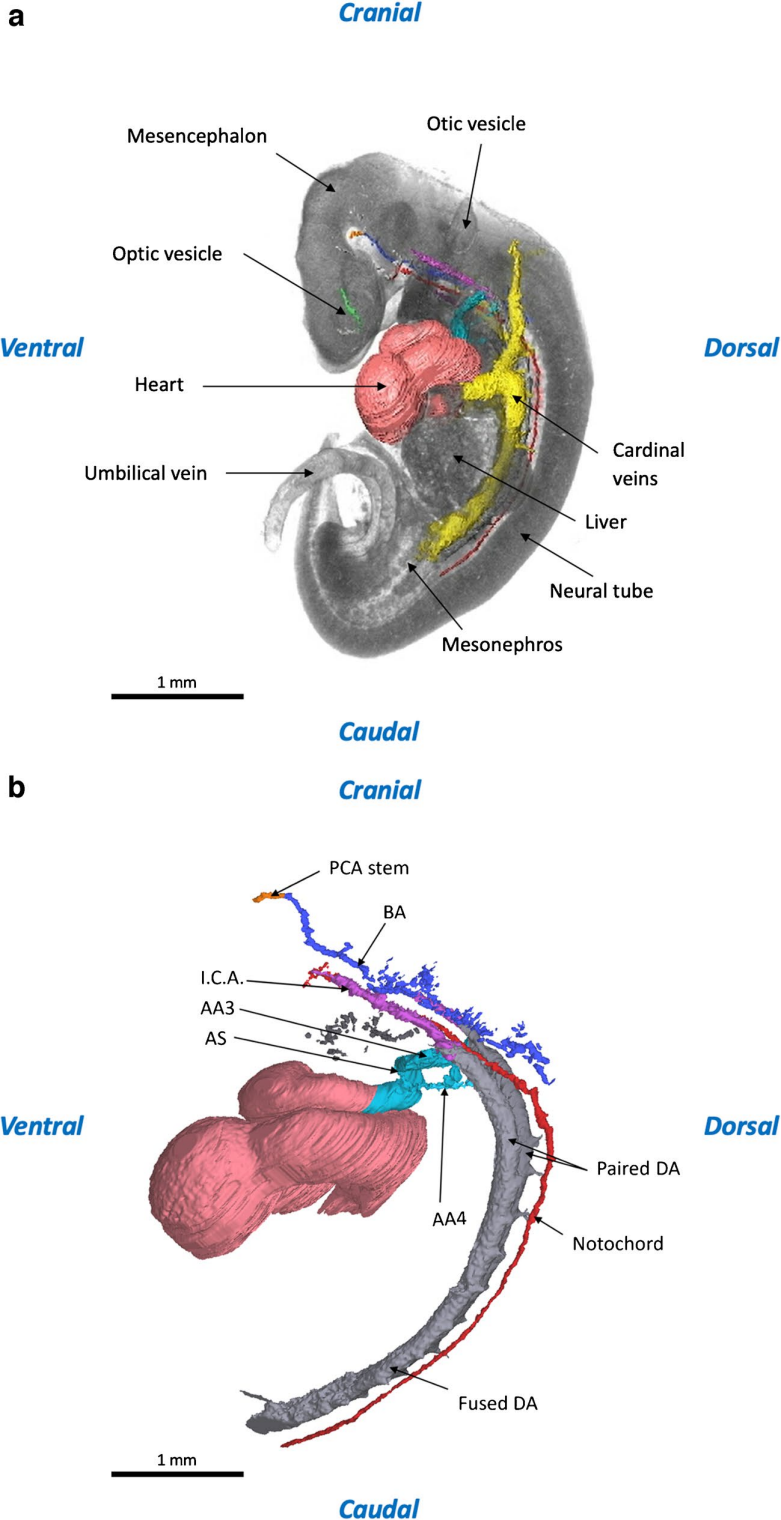
Three-dimensional reconstruction at Carnegie stage 14 (4-mm crown-rump length). **a** In situ 3-dimensional reconstruction of the intra- and extracranial vascular system. **b** Vascular tree 3-dimensional reconstructions of the intra- and extracranial vascular system. Panels (**a**) and (**b**) were both generated from micro-computed tomography contrast-enhanced high-resolution images (2.5-µm voxel size); the structures shown are listed in Table 3. *AA3* third pharyngeal arch artery, *AA4* fourth pharyngeal arch artery, *AS* aortic sac, *BA* basilar artery, *DA* dorsal aorta, *ICA* internal carotid artery, *PCA stem* posterior cerebral artery

When examining our 3-D reconstruction of a human embryonic specimen at CS 14, several unique observations were made. Our reconstruction revealed numerous cardiovascular structures, including the posterior cerebral artery (PCA stem), basilar artery (BA), internal carotid artery (ICA), third pharyngeal arch artery (AA3), fourth pharyngeal arch artery (AA4), aortic sac (AS), and dorsal artery (DA). Clearly in this stage of development, we have not been able to annotate and reconstruct all arterial structures. Some arterial structures that according to literature should have been visible were not present or visible in our data set (Table 3). The third and fourth pharyngeal arches are perceptible and bilaterally connected to the aortic sac. Figure 3 presents the cardiovascular anatomy from our reconstruction in situ, and Table 3 demonstrates an overview of the cardiovascular structures presented in our reconstruction compared with literature.

**Table 3:**
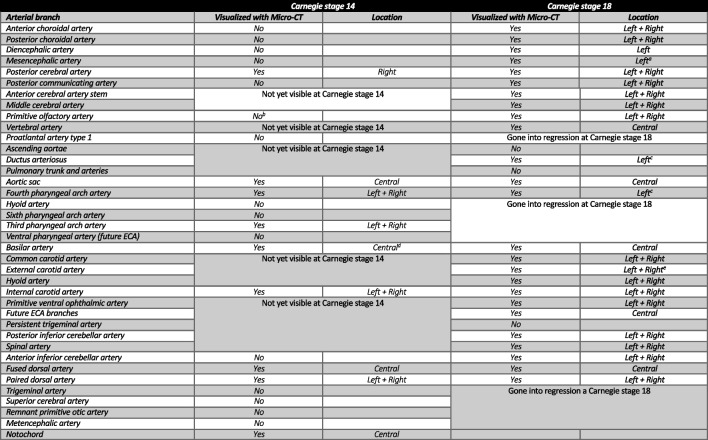
Overview of our findings from the reconstructions of human embryonic specimen from Carnegie stages 14 and 18. Visualized “Yes” and non-visualized “No.” List of arteries is based on our findings in literature. The list of structures should be present or visible at Carnegie stages 14 and 18 according to literature [27]

^a^The mesencephalic artery was partially visible. ^b^Starts to develop but there is no visible continuity yet. ^c^These structures are anatomically left-sided. ^d^Is fusing in this stage of development. ^e^The right external carotid artery is not fully connected to the common carotid artery in the 3-dimensional construction. *ECA* external carotid artery

### Reconstruction embryonic sample 2, CS 18

During our examination of the 3-D reconstruction of a human embryonic specimen at CS 18, we identified several significant anatomical features. The posterior inferior cerebellar arteries and the spinal arteries were nicely visible. Moreover, our reconstruction displayed detailed branches from the hyoid artery and the anterior choroidal artery. The external carotid artery was also well-represented, showing multiple vascular branches. Furthermore, we demonstrate various vascular structures in detail. The vertebral arteries displayed a noticeable zig-zag configuration, the basilar artery showed intricate side branches, and the primitive central ophthalmic artery extended to the eye. Certain structures were exclusively discernible on the left side of the embryo, including the diencephalic artery, the mesencephalic artery, and a part of the external carotid artery. However, some cardiovascular structures were not observable in our reconstruction. These included the persistent trigeminal artery, the ascending aortae, the pulmonary trunk, and the pulmonary arteries.

**Fig. 4:**
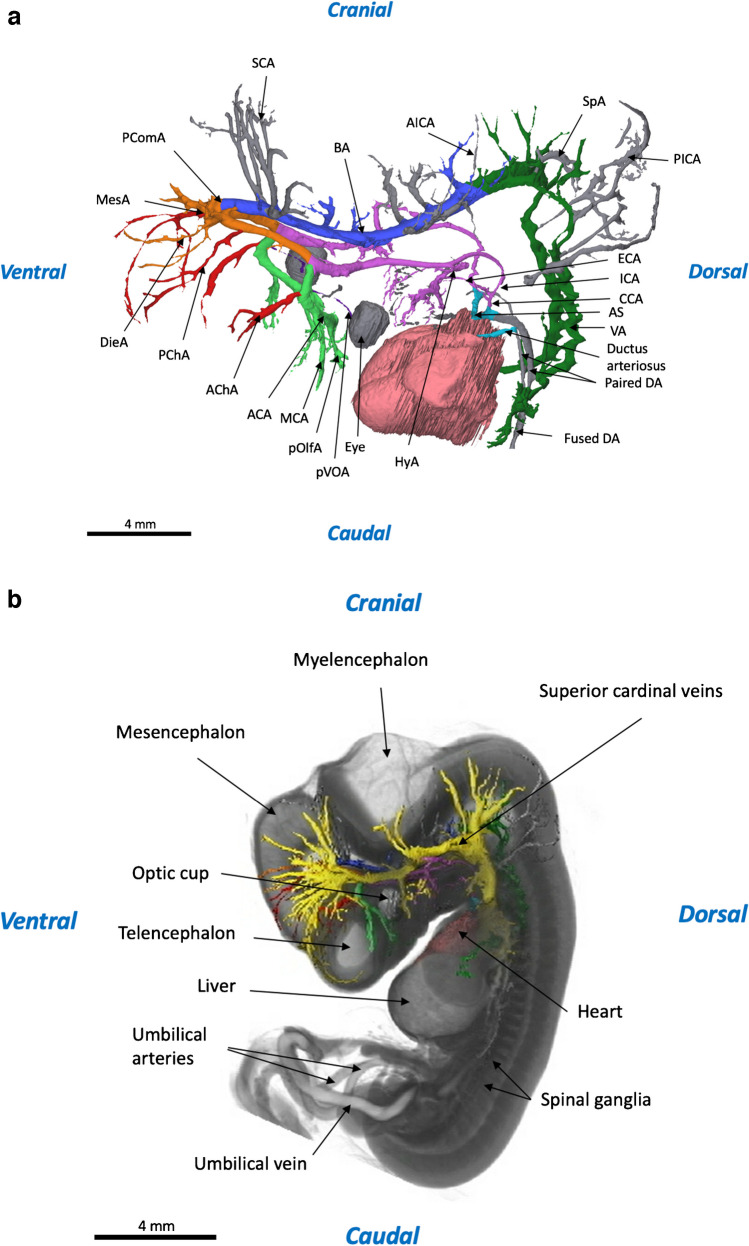
Three-dimensional reconstruction at Carnegie stage 18 (15-mm crown-rump length). **a** In situ 3-dimensional reconstruction of the intra- and extracranial vascular system. **b** Vascular tree 3-dimensional reconstructions of the intra- and extracranial vascular system. Panels (**a**) and (**b**) were both generated from micro-computed tomography contrast-enhanced high-resolution images (9.0-µm voxel size); the structures shown are listed in Table 3. *ACA* anterior cerebral artery, *AChA* anterior choroidal artery, *AICA* anterior inferior cerebral artery, *AS* aortic sac, *BA* basilar artery, *CCA* common carotid artery, *DA* dorsal aorta, *ECA* external carotid artery, *HyA* hyoid artery, *ICA* internal carotid artery, *MCA* middle cerebral artery, *PCA stem* posterior cerebral artery, *PChA* posterior choroidal artery, *Pcom* posterior communicating artery, *PICA* posterior inferior cerebral artery, *P.olf.A* primitive olfactory artery, *PVOA* primitive ventral ophthalmic artery, *SpA* spinal artery, *VA* vertebral artery

Figure 4 presents the cardiovascular anatomy from our reconstruction, schematic and in situ, and Table 3 demonstrates an overview of the cardiovascular structures presented in our reconstruction compared with literature.

## Discussion

Our aim was to investigate the craniofacial vascular development in early-stage human embryos, with a particular focus on extracranial arteries. This topic has been underrepresented in current literature, despite the critical importance of early embryonic stages in laying the foundation for eventual anatomy. Through this research, we hope to contribute to a better understanding of congenital anomalies in the craniofacial region, such as craniofacial synostoses, cleft lip and cleft palate, micrognathia, and macroglossia [2–4].

This study marks the first instance of non-destructive micro-CT imaging being used to chart the craniofacial structures of early human embryos. Generally, our results align with sources that describe embryonic development via histology [27, 41]. Specifically, in CS 18, we delved deeper into detailing the branches of intra- and extracranial arteries through the creation of 3-D reconstructions.

For educational purposes, we have rendered the 3-D reconstructions of the two embryos from this pilot study into 3-D-PDF format (supplementary materials). This allows readers to interactively explore the models, identifying and toggling individual structures, enhancing students’ 3-D understanding of complex vascular structures [42].

### Unique embryonic samples

The Dutch Fetal Biobank, established in 2017 at Amsterdam UMC, is a unique resource for embryonic and fetal tissue [43]. Its uniqueness stems from three factors: the liberal political climate in The Netherlands essential for such a resource’s existence, high parental willingness to donate to science, and the biobank team’s round-the-clock readiness to collect donations, ensuring tissue is included within hours of birth. Additionally, unlike many countries where pregnancies are terminated via curettage, The Netherlands increasingly prefers medical terminations due to the adverse effects of curettage on future pregnancies [44]. This preference ensures we receive intact specimens, facilitating thorough anatomical research.

The two embryos used in this study were obtained through the surgical removal of a fallopian tube in ectopic pregnancies, where the pregnancy implants in a non-uterine location, posing a life-threatening risk to the mother. A question arises whether this abnormal location impacts the embryo’s development, potentially due to suboptimal placental development [45]. However, our experience in studying human embryos from ectopic pregnancies indicates their development aligns with the known Carnegie staging, and their anatomy is comparable to intrauterine embryos. The impressive vascularization around the fallopian tube may ensure adequate placental development during early stages. Despite our experience and the similarities in Carnegie staging and anatomical comparability, we must acknowledge that there is no absolute certainty that extrauterine and intrauterine developments are identical. Therefore, we must be cautious when interpreting data obtained from ectopic pregnancies, as it may not be equivalent to that from intrauterine embryonic development.

This pilot study’s inclusion of only two human embryos is insufficient to draw conclusions about anatomical variation. Early-stage human embryonic samples are extremely rare, but with the growing number of samples in the Dutch Fetal Biobank, we aim to provide a comprehensive overview of craniofacial vascular development. We hypothesize that alterations in vascular development can negatively impact normal growth and development, potentially contributing to orofacial malformations. Further research is needed to substantiate the role of vascular development as an etiological factor for these malformations. Understanding the normal development of cranial arteries will enable comparisons with aberrant developmental patterns in embryos with congenital anomalies currently being prospectively collected in the biobank.

### Micro-CT imaging as a method to study vasculature

Historically, mapping craniofacial arteries in human embryos required destructive methods, including histology, immunohistochemistry, and light sheath microscopy [9, 10, 16]. Given the scarcity of complete and healthy human embryos available for scientific research, it is imperative to handle these specimens with the utmost respect, ideally preserving them intact. Our experience with contrast-enhanced micro-CT imaging using buffered Lugol’s solution to prevent extensive shrinkage (ranging from 15 to 35% for lung, brain, liver, or total body volume when using non-buffered Lugol’s solution) has enabled us to scan embryos obtained from ectopic pregnancies while maintaining the integrity of the amniotic sac, at resolutions of 2.5-μm or 9-μm voxel size. For perspective, the average human hair is 70 μm thick. In this pilot study, we presented results from the first two human embryos mapped using micro-CT imaging.

Our findings revealed that vascular structures in our models sometimes lacked bilaterality, contrary to expectations for these developmental stages. A plausible explanation, supported by histological studies of human embryos [27, 41], is the post-mortem redistribution of blood cells to the lowest side when an embryo is laid down. This phenomenon which is also known from adult post-mortem cases likely occurs before fixation, making vessels on the lower side appear fuller than those on the higher side. The higher side’s small vessels sometimes seem “empty” as they contain no red blood cells. Combined with iodine contrast’s high affinity for blood, this makes reconstructing vessels on the higher side challenging or impossible.

Another challenge of our method is the manual segmentation required for annotating all vessels in micro-CT images. This is an established method for studying anatomical structures in 3-D, with low inter-observer variability when performed by trained yet unbiased students under experienced embryologists’ supervision [41]. The relative standard error in such manual reconstructions ranges from only 0.3% for simple structures to 2% for complex ones such as blood vessels [27]. However, manual segmentation is time-intensive, and advances in artificial intelligence may soon offer solutions to this issue [5].

## Conclusion

Our study presents the first non-destructive mapping of craniofacial arteries in early-staged human embryos using micro-CT imaging, revealing valuable insights into vascular development and providing educational models. Although limited by the small sample size, our findings lay the groundwork for future research with larger cohorts from the Dutch Fetal Biobank, ultimately advancing our understanding of normal and abnormal craniofacial vascular development.

## Supplementary information

Below is the link to the electronic supplementary material.

Supplementary file1 (PDF 6682 KB)

Supplementary file2 (PDF 11017 KB)

## Data Availability

The data that support the findings of this study are available from the corresponding author, K.J., upon reasonable request.
