# Supplementary figures and images for: Imaging the development of the human craniofacial arterial system – an experimental study
Source: Pediatr Radiol. 2024 Sep 10;55(4):721–32. doi: 10.1007/s00247-024-06044-x (PMC11982102; doi:10.1007/s00247-024-06044-x)

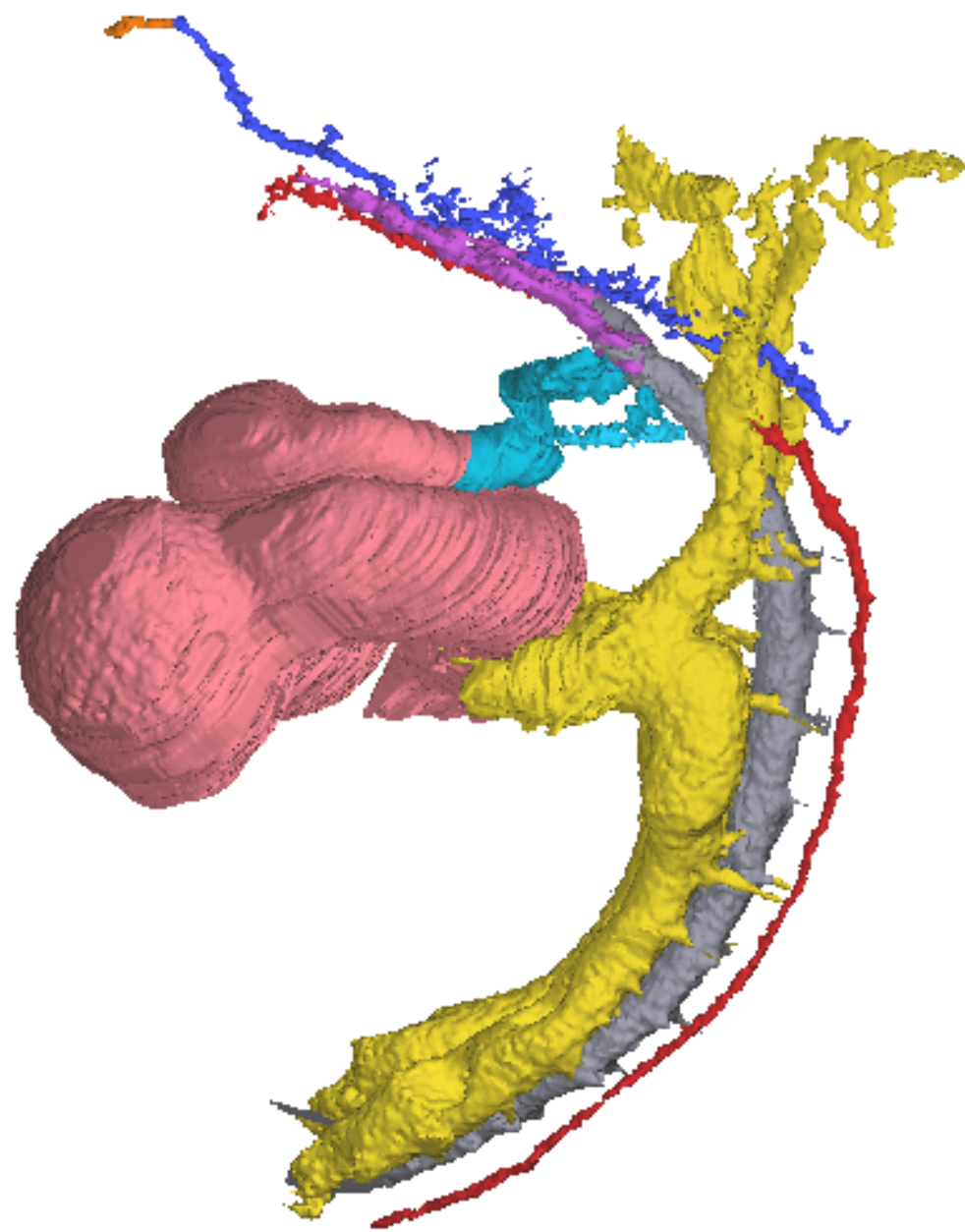

Supplement: Supplementary file 1 — Supplementary file1 (PDF 6682 KB) [file 247_2024_6044_MOESM1_ESM.pdf]

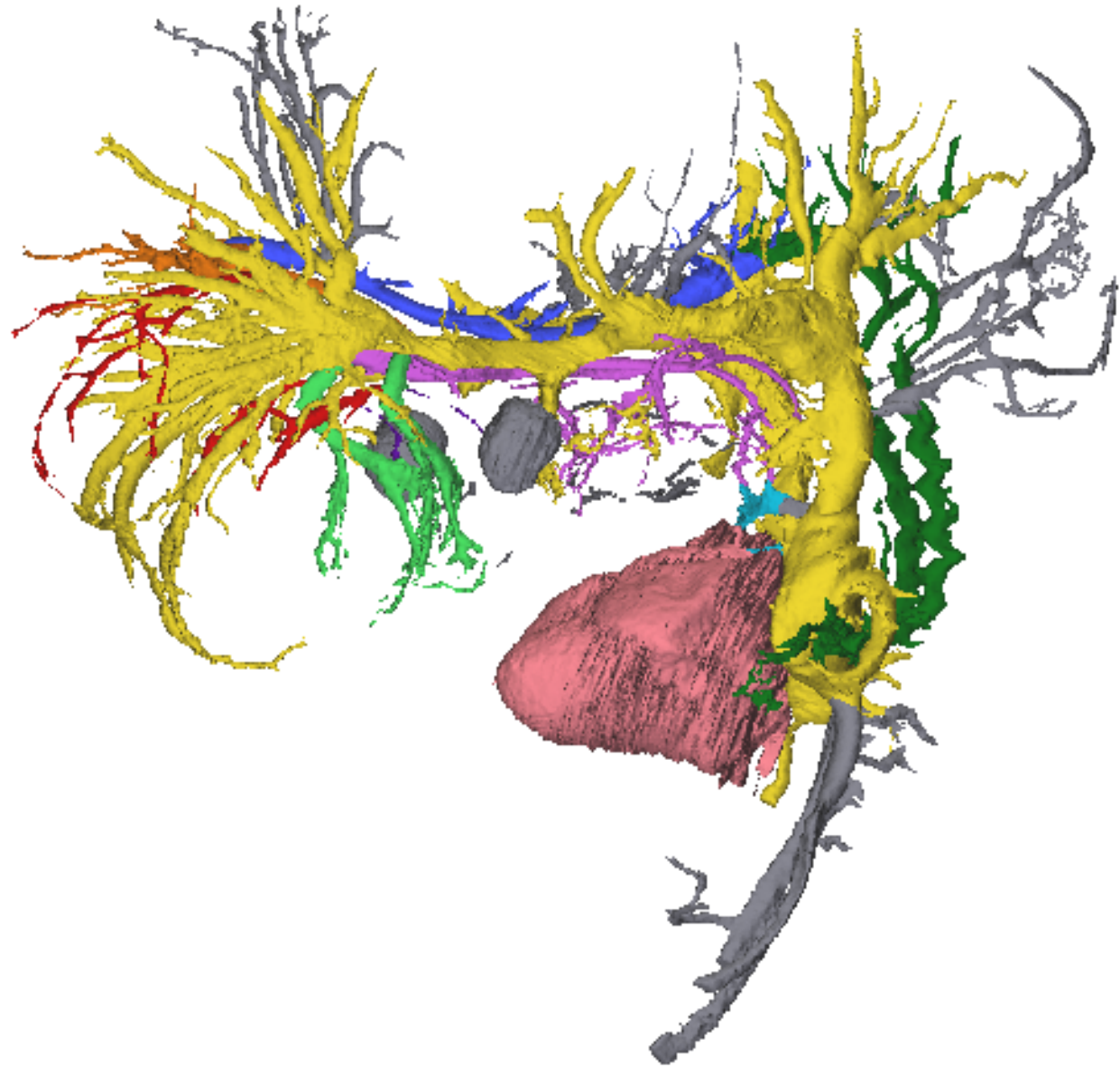

Supplement: Supplementary file 2 — Supplementary file2 (PDF 11017 KB) [file 247_2024_6044_MOESM2_ESM.pdf]
